# Effect of combined use of supplementary irrigation, manure and P fertilization on grain yield and profitability of soybean in northern Nigeria

**DOI:** 10.1016/j.heliyon.2024.e28749

**Published:** 2024-03-25

**Authors:** J.F. Bebeley, A.Y. Kamara, J.M. Jibrin, A.I. Tofa, R. Solomon, N. Kamai

**Affiliations:** a International Institute of Tropical Agriculture (IITA), Kano, Nigeria; b Current Address: Sierra Leone Agricultural Research Institute, Freetown, Sierra Leone; c Centre for Dryland Agriculture, Bayero University, Kano, 700241, Nigeria

**Keywords:** Agroecology, *Bradyrhizobium*, Phosphorus, Moisture-stress, Input-bundle, Nodumax

## Abstract

Declining soil fertility particularly phosphorus deficiency, low organic carbon, moisture stress and high cost of input are factors limiting soybean yield in the Nigeria savanna. Supplementary irrigation, nutrient application and inoculation with *Bradyrhizobium* could increase the grain yield of soybean. We evaluated the effects of Rhizobia inoculant, phosphorus fertilization, manure, and supplementary irrigation on the nodulation and productivity of a tropical soybean variety in two locations in northern Nigeria in the 2017 and 2018 cropping seasons. The treatments consisted of five input bundles: Supplementary irrigation +17.5 kg P ha^−1^ + 4 t ha^−1^ poultry manure + nodumax inoculant (S + P + M + I); 17.5 kg P ha^−1^ + 4 t ha^−1^ poultry manure + nodumax inoculant (P + M + I); 17.5 kg P ha^−1^ + nodumax inoculant (P + I); 17.5 kg PP ha^−1^ (P); and nodumax inoculant (I). Economic analysis was done to determine the benefit-cost ratio (BCR) for each input bundle. In Kano, the input bundle S + P + M + I produced mean number of nodules that were 38, 102, 200 and 352% higher than that of input bundles P + M + I, P + I, P and I, respectively. At Lere, the application of input bundle S + P + M + I increased mean number of nodules by 33, 81, 93 and 182% over that of input bundles P + M + I, P + I, P and I, respectively. Mean grain yield in Kano was greater for input bundle S + P + M + I over P + M + I, P + I, P and I bundles by 31, 50, 64 and 223%, respectively. In Lere, grain yield for input bundle S + P + M + I was higher than that of input bundles P + M + I, P + I, P and I only, by 27, 47, 41 and 184% respectively. The input bundle P + M + I produced the highest BCR (1.4) in Kano and application only of P produced the highest BCR (1.3) in Lere. Supplementary irrigation was not found to be profitable due to the high cost of supplementary irrigation.The application of P with or without manure/inoculant is recommeded for profitable soybean production in the savannas of Nigeria.

## 1 Introduction

The cultivation of soybean (*Glycine max* [L.] Merr.) is increasing in the savannas of Nigeria, and it has become a major cash crop widely used in the food and feed industry [1]. Soybean farmers in Nigeria are now aware of its potential as cash or food crops hence increasing their production to improve income through domestic and export markets [2]. Due to the availability of soybean produced in northern Nigeria, the region has experienced an increase in the number of industries especially oil mills, livestock feed mills, and cereal processing industries [2]. The availability of soybean meal in Nigeria has helped in the rapid growth of Nigeria's poultry industry [3]. The production of soybean has also helped to attain food security, reduce poverty among farmers and improve the nutritional status of Nigerians [2].

Nigeria is currently the 16th largest soybean producer globally, and after South Africa, the second largest in Africa. Soybean production has increased from 73,000 t from the 250,000 ha harvested in 1979 to 600,000 t from 647,292 ha in 2020 [4]. Despite this importance, poor soil fertility, moisture stress due to erratic rainfall and high cost of required inputs limits productivity [[5], [6], [7]] in the soybean producing areas in the Nigeria savannas. Agricultural production is mainly under resource-constrained and rain-fed farming systems on poor soils in the savanna regions of Nigeria [8]. Low annual precipitation, drought, high evapotranspiration, and very high inter and intra-seasonal rainfall variations worsen the situation [[9], [10], [11]]. The average yield of soybean is less than one ton ha^−1^, which is below the potential yield of over 3 tons ha^−1^ [12]. The soils of the savana regions are primarily sandy and often prone to erosion with very low organic matter, water-holding capacity, and nutrient contents [13,14]. Phosphorus contents are below the critical levels (10–15 mg kg^−1^) required for soybean production in savanna soils [15]. Because of very low levels of soil-available phosphorus and organic matter, soybean yield is limited [7,16,17]. Phosphorus deficiency is the most important constraint that affects the growth and yield of grain legumes such as soybean [16,18,19]. Kamara et al. [19] reported soybean yield reduction of 1314 kg ha^−1^ when no P was applied compared to the application of 40 kg P ha^−1^. Applying phosphorus (P) fertilizer can overcome P deficiency on soils that do not strongly adsorb it [20]. The availability of P improves nodulation and increases biological nitrogen fixation which promotes the growth of different plant organs [18]. Lack of soil organic carbon limits the effectiveness of other nutrients in the soil [21], therefore, its sustenance is very important in maintaining soil fertility [22]. Improving the carbon content of the soil through the addition of organic manure has the tendency to improve the response of soybean to rhizobia inoculation and P-fertilizer [23]. According to Chiezey and Odunze [24], the application of 1 t ha^−1^ of poultry manure increased grain yield of soybean by 33.7% compared with plots without manure.

Soybean, like many other crops, is negatively affected by moisture stress caused by intermittent drought leading to low yield in soybean production [25]. Cultivation of soybean in Nigeria is predominantly under rainfed agriculture where rainfall is increasingly becoming erratic and can cause water stress and significant loss of yield [26]. Many Authors have reported yield reduction in soybean as a result of moisture stress. For example, Mustapha et al. [27] reported grain yield loss of 53.9 and 60.7% at vegetative and pre-flowering, respectively, and 19.6% at post-flowering stages as a result of moisture stress of soybean in a pot experiment conducted at Ilorin in northern Nigeria. Rise in global temperatures and changing precipitation patterns pose a significant threat to soybean production, especially in rainfed areas [28,29]. The use of early maturing variety and supplementary irrigation are climate smart strategies for drought adaptation as soybean can be considered the best crop for adapting to climate change due to the available market for the farmers [30]. With the huge impacts of climate variability on small-holder farmers in developing countries, climate-smart soybean production has the potential to boost the income level of small-holder farmers [30]. Supplementary irrigation could therefore serve as a strategy in adapting to climate change and population pressure, particularly in African countries where food security is highly vulnerable [31,32]. Several reports have indicated that supplementary irrigation could increase soybean grain yield under rainfed agriculture [33,34].

Other constraints are the high costs of inputs such as fertilizer, inoculants, herbicides and pesticides [35]. Although many farmers in Nigeria use fertilizers, most is applied to maize and at rates well below what is recommended [36,37]. With respect to soybean production, the acquisition of inputs such as fertilizer and agro-chemicals may be difficult for farmers, thereby leading to low productivity [38]. The high cost of fertilizer makes it unaffordable for farmers resulting into low use of fertilizers and low yields due to depleted fertility of the soil. The purchase of fertilizer also increases cost of production thereby increasing total variable cost and reducing profit in soybean production [38].

Several endeavors have been made to improve soybean yields in smallholder farms through combined application of inoculant with appropriate rhizobia strains and P fertilizer [5,12,23]. However, few studies have attempted to evaluate the response of soybean to the combined application of rhizobium inoculant and P fertilizer under supplementary irrigation. While several studies have evaluated the profitability of the combined application of rhizobia inoculant and P fertilizer in improving soybean grain yield in smallholder farms, few studies have assessed the profitability of the combined application of these inputs under supplementary irrigation. Good returns on investment for their productive efforts have an influence on farmers, but increasing crop yields require investment in productivity to produce profit and allow them to save and reinvest in other activities [5]. The use of combined inputs in production gives smallholder farmers an opportunity for higher grain yield and positive returns [39]. Rao and Reddy [40] reported a minimum Benefit-Cost-Ratio (BCR) ≥1 as profitable for adoption of input in soybean. In northern Ghana, Ulzen et al. [23] reported BCR ≥1 with the combined application of Rhizobia inoculant, P-fertilizer, and organic manure in a soybean inoculation trial.The objective of the study was to evaluate the response of soybean to combined application of nutrient inputs under supplementary irrigation and to assess the profitability of the combined use of these input under supplementary irrigation. This information will guide smallholder soybean farmers towards more profitable soybean production in the Guinea savannas of Nigeria.

## 2 Materials and methods

### 2.1 Description of study area

**Fig. 1:**
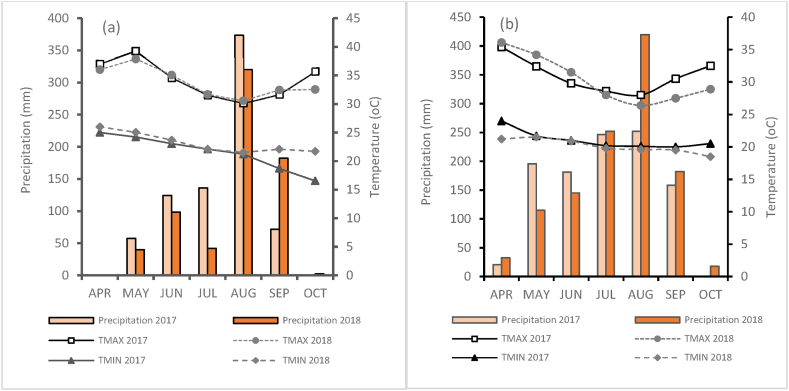
Monthly mean, minimum, and maximum temperatures, and total rainfall in Kano (a) and Lere (b) during the 2017 and 2018 growing seasons.

A field study was conducted during the 2017 and 2018 growing seasons; June–October in two locations: Kano (11°58.861′N, 008°25.155′E, 449 m asl) in the Sudan savanna (SS) and Lere (10°30.466′N, 008°31.177′E, 784 m asl) in the Northern Guinea savanna (NGS). Soybean performs well in the NGS where rainfall is more than 700 mm. The early maturing variety used in this study (TGX1987-62F), when sown early, can thrive in the SS where rainfall duration is short. The climate of the two sites is typical of the West African savannas characterized by a long dry season followed by a rainy season that extends from May to October. The average annual precipitation in Kano was 763 mm in 2017 and 685 mm in 2018, with an average maximum temperature of 33.8 °C and a minimum of 21.0 °C (Fig. 1a). In Lere, the average annual precipitation was 1054 mm in 2017 and 1165 mm in 2018, with an average maximum temperature of 31.0 °C and a minimum of 21.1 °C (Fig. 1b). Composite samples of top soils (0–15 cm) were taken from the experimental sites with an auger before planting and fertilizer application and analyzed for soil texture, pH, organic carbon, total N, available P and Exchangeable K [41].The soil type was Plinthic Lixisols at the experimental site of Bayero University Kano, and Haplic Lixisols at Lere, according to World Reference Base for Soil Resources [42]. The soil in Kano was a sandy loam (665 g kg^−1^ sand, 165 g kg^−1^ silt, and 170 g kg^−1^ clay) with low levels of organic carbon (7.9 g kg^−1^), total N (0.65 g kg^−1^), and available P (5.64 mg kg^−1^). The Exchangeable K content was moderate (0.23 cmol kg^−1^) with a moderately acidic pH (6.0). The soil in Lere was sandy clay loam (410 g kg^−1^ sand, 280 g kg^−1^ silt, and 210 g kg^−1^ clay) with low organic carbon (8.5 g kg^−1^), moderate total N (1.15 g kg^−1^), and low available P (6.92 mg kg^−1^). The K content (0.27 cmol kg^−1^) was moderate with pH of 5.3. The classification of soil properties was adopted from Esu [43] classification of tropical soils.

### 2.2 Treatment and experimental design

The variety TGX1987-62F (an early maturing variety with 90–100 days to maturity developed by IITA) was used at both sites. The treatments consisted of five input bundles: Supplementary irrigation +17.5 kg P ha^−1^ + 4 t ha^−1^ poultry manure + nodumax inoculant (S + P + M + I) [3,44]; 17.5 kg P ha^−1^ + 4 t ha^−1^ poultry manure + nodumax inoculant (P + M + I); 17.5 kg P ha^−1^ + nodumax inoculant (P + I); 17.5 kg P ha^−1^ only (P); nodumax inoculant only (I). The experiments were replicated three times in a randomized complete block design (RCBD). The plots consisted of four rows 5 m long with 75 cm between rows and 10 cm between plants within a row. Eight seeds were planted at a depth of about 5 cm and later thinned to four plants per stand at 2 weeks after planting (WAP), giving a plant density of 533,333 plants ha^−1^. When supplementary irrigation treatment was necessary to keep the plots well-watered, pipes supplied water through the furrows between the ridges until the plots reached field capacity. In plots with the P treatment, 17.5 kg P ha^−1^ in the form of Triple Superphosphate fertilizer was basally applied by banding on the top of the ridges at planting. For treatments containing manure, poultry manure was applied at the rate of 4 t ha^−1^ before planting. The poultry manure contained 3.8% N, 3% P, 1.7% K and 30% organic carbon. For treatments containing inoculants, soybean seeds were inoculated using nodumax, containing *Bradyrhizobium japonicum* strain USDA110 (2 × 109 viable cells g^−1^) [45]. About 10 g of the enclosed packet of gum Arabic was dissolved in 100 mL of warm water. Soybean seeds (5 kg) were poured into a large basin; the sticker was added and the seeds were mixed until uniformly coated. Fifty grams of the nodumax inoculant containing *Bradyrhizobium* bacteria was added and mixed until the seeds were uniformly covered with the inoculant. The inoculated seeds were covered with a cloth and the sticker was allowed to rest for 10 minute. The inoculated seeds were planted in a moist seedbed.

### 2.3 Cultural practices

The land was prepared by spraying the fields with glyphosate at 4 L ha^−1^. It was then harrowed and ridged three weeks after spraying before the experimental plots were laid out. In 2017, seeds were sown on 4 July in Kano and on 21 June in Lere; in 2018, sowing was done on 28 June in Kano and 30 June in Lere. At planting, each plot received a basal application of 33.1 kg K ha^−1^ as muriate of potash [44]. Immediately after sowing, a combination of pendilin (500 g L^−1^ pendimethalin) and gramaxone (1:1-dimethyl-4,4-bipyridinum dichloride) were applied at the rate of one L ha^−1^ using a knapsack sprayer. This was followed by hand weeding three weeks later to control subsequent weeds.

### 2.4 Measurements

The crop data collected during the experimental periods included the number of nodules, total dry matter, and grain yield. The number of nodule was determined at full flowering. Five plants were randomly sampled from the border rows for nodule count determination. Soybean plants were carefully dug out using a shovel. The roots were separated from the shoots and washed with clean water to remove adhering soil. The nodules were carefully detached from the roots and counted [46].

The two middle rows were used for the determination of dry matter and grain yield. At maturity, all the plants in a quadrat measuring 1.5 m^−2^ and placed across the two middle rows were pulled for the measurement of total dry matter and grain yield. Leaves, stems, and pods were oven-dried to a constant weight at 60 °C for 76 h in a forced-draft oven and weighed [18]. The weights were added together to obtain the total dry matter in gm^−2^ which was converted to kg ha^−1^. For grain yield determination, pods on the remaining plants from the two middle rows were harvested, sun-dried, and hand-threshed. The grain from the two middle rows was combined with grain from the quadrat and weighed to obtain total grain yield. The moisture content of grain samples was determined using Farmex grain moisture tester (Farmex MT-16, Farmcomp Oy, Tuusula, Finland) before being weighed. Grain weight was adjusted to 12% moisture content and expressed as kg ha^−1^.

### 2.5 Economic analysis

The economic analysis was conducted by estimating BCR which defines the amount of additional output from a unit increase in nutrient input [40,47]. The variable costs of inputs (seed, inoculum, TSP, and supplementary irrigation), with labour for agronomic management in planting, weeding, P application, irrigation, harvesting, and threshing were actual market prices from the experimental farm records averaged over the 2‐year production period. Human labour was calculated as person‐day ha^−1^ on 8 h, equivalent to one person‐day output. All inputs and outputs were summarized to determine the cost of production. Grain yield (kg ha^−1^) was multiplied by prevailing market prices at harvest to determine gross returns for each treatment. Stover yield was not calculated as part of the economic output. The output price was quoted as farmgate prices in 2017 and 2018. All prices were recorded in local currency (Nigerian Naira - ₦) and converted into US dollars (US$ 1 = ₦350) currency exchange rate. Economic analysis was calculated using the formula;

$$BCR=\frac{MGR-MVC}{MVC}$$

Where; BCR is the benefit-cost ratio, MGR is the mean gross return, and MVC is the mean variable cost (adopted from Awuni et al. [5]). A BCR >1 mean a net profit while <1 means a net loss. The higher the BCR, the more attractive the use of inputs [40].

### 2.6 Statistical analysis

Data collected from the experiment was tested for normality as an important analysis of variance (ANOVA) assumption through the relationship between the range and standard deviation [48]. Numerical approach was used to test for the normality of the data. Levene's test for homogeneity of variances [49] was used to examine the plausibility of homoscedasticity for the data. After testing the data for normality and homogeneity of variances, one-way ANOVA was performed using PROC GLM in Statistical Analysis System (SAS) software version 9.4 [50]. Treatments were considered as fixed effects and replications were random effects. Means were determined for treatments; comparisons were done at p ≤ 0.05 significance level using Least Significant Difference (LSD).

## 3 Results

### 3.1 Effect of location and year on crop growth and yield

Location and year had significant effects on all the measured parameters (Table 1). Mean number of nodules, total dry matter and grain yield were higher in Kano than in Lere. The number of nodules produced at Kano was 29% higher than that produced at Lere. Similarly, the total dry matter and grain yield were 21% and 18% higher, respectively in Kano, than in Lere. The measured parameters also differed between the years. The mean number of nodules, total dry matter, and grain production were all higher in 2018 compared to 2017. The number of nodules was 30% higher in 2018 than in 2017. Total dry matter and grain yield increased by 17% and 13%, respectively, in 2018 compared to 2017. There was signficant interaction between year and location for number of nodules, total dry matter, and grain yield. In 2017, the number of nodules produced in Lere was not signficantly different from that of Kano while in 2018, the number of nodules was significantly higher in Kano than in Lere. Total dry matter and grain yield were generally higher in Kano than Lere for both years, but the magnitude of the differences between the locations was higher in 2017 than in 2018.

**Table 1:** Interaction effect of location and year on number of nodules, total dry matter, and grain yield of soybean at Kano and Lere in 2017 and 2018 rainy seasons.

	Number of nodules plant^−1^			Total dry matter (kg ha^−1^)			Grain yield (kg ha^−1^)		
Location (L)/Year (Y)	2017	2018	Mean	2017	2018	Mean	2017	2018	Mean
Kano	58	121	89	7473	8079	7776	2166	2292	2229
Lere	67	59	63	5124	7104	6114	1608	2030	1819
Mean	63	90		6298	7592		1887	2161	
LSD_5%_ L	10.6**			495.6**			112.2**		
LSD_5%_ Y	11.3**			498.2**			114.9**		
LSD_5%_ L × Y	15.0**			700.8**			158.8*		

LSD = Least significant differences, ** and * = Significant at 1 and 5 % levels of probability, respectively.

### 3.2 Effect of input bundles on nodulation

Table 2 shows the effect of input bundles on number of nodules. The input bundle containing the application of supplementary irrigation, phosphorus, and manure along with inoculation with *Bradyrhizobium* (S + P + M + I) consistently increased the number of nodules compared with the other input bundles in both locations. In the two study areas, treatment I (only *Bradyrhizobium*), produced the lowest number of nodules and was comparable to the application of treatment P. In Kano, the input bundle S + P + M + I in 2017 produced number of nodules that were 54, 110, and 218%, respectively, more than those of the bundles P + M + I, P + I, and P. The combined application of phosphorus, manure, and Rhizobia inoculant without supplementary irrigation (input bundle P + M + I) produced number of nodules that were 36 and 106% more than those from the bundles P + I and P. The application of input bundle P and the Rhizobia inoculant (P + I) gave a number of nodules that was 52% higher than that of input bundle P alone. In 2018, input bundle S + P + M + I produced number of nodules that were 31, 99, and 193% more than those of P + M + I, P + I, and P, respectively. The application of input bundle P + M + I produced number of nodules that were 52 and 123% higher than those of input bundles P + I and P. The difference between input bundles P + I and P in number of nodules was not significant.

**Table 2:** Effect of input bundles on number of nodules plant^−1^ of soybean at Kano and Lere in 2017 and 2018 rainy seasons.

Treatments	Kano		Lere	
	Number of nodules plant^−1^		Number of nodules plant^−1^	
	2017	2018	2017	2018
S + P + M + I	103.3^a^	218.8^a^	100.9^a^	92.0^a^
P + M + I	67.0^b^	166.7^b^	73.4^b^	71.9^b^
P + I	49.3^c^	109.8^c^	62.8^b^	43.9^c^
P	32.5^d^	74.8^cd^	58.6^bc^	41.3^c^
I	38.0^cd^	33.3^d^	35.1^c^	33.4^c^
LSD	11.96	51.52	26.68	18.07
P value	<0.0001	0.0008	0.0042	0.0001
CV	46.5	61.9	37.4	43.0

I: Inoculant; P: Phosphorus; M: Manure; S: Supplementary irrigation. Within each.

Location and for each variable, means followed by the same letter are not significantly.

Different according to LSD (P ≤ 0.05).

In Lere, input bundle S + P + M + I produced number of nodules in 2017 that were 37, 61, and 72% more than those of input bundles P + M + I, P + I, and P, respectively. There were no significant differences among input bundles P + M + I, P + I, and P in terms of number of nodules. In 2018, the application of input bundle S + P + M + I in Lere produced number of nodules that were 28, 110, and 123% more than those of input bundles P + M + I, P + I, and P, respectively. The application of input bundle P + M + I produced number of nodules that were 64 and 74% more than those of input bundles P + I and P. The difference was not significant (Table 2) between input bundles P + I and P for number of nodules.

### 3.3 Effect of input bundle on total dry matter and grain yield

The application in 2017 at Kano of input bundle S + P + M + I increased total dry matter by 24, 22, and 89% over that of input bundles P + I, P, and I, respectively (Table 3). The input bundle S + P + M + I produced dry matter at Kano that was statistically similar to that of input bundle P + M + I. The application of input bundle P + M + I increased total dry matter by, 15, 14, and 76% compared with input bundles P + I, P, and I, respectively. The input bundle P + I increased total dry matter by 53% over I. The difference between input bundles P + I and P was not significant. The application of P increased total dry matter by 55% more than I (Table 3). In 2018, the combined application of supplementary irrigation, P, and manure along with inoculation with *Bradyrhizobium* increased total dry matter by 109% over I at Kano. There were no significant differences among the four input bundles S + P + M + I, P + M + I, P + I, and P. The application of input bundle P + M + I increased total dry matter by 81% compared with I. The application of input bundle P + I increased total dry matter by 76% over I. The difference in 2018 between input bundles P and I was not significant.

**Table 3:** Effect of input bundles on total dry matter of soybean at Kano and Lere in 2017 and 2018 rainy seasons.

Treatments	Kano		Lere	
	Total dry matter (kg ha^−1^)		Total dry matter (kg ha^−1^)	
	2017	2018	2017	2018
S + P + M + I	9151.3^a^	10346.9^a^	7417.9^a^	8114.2^a^
P + M + I	8500.6^a^	8961.6^a^	5335.6^b^	8179.0^a^
P + I	7390.9^b^	8700.1^a^	4684.9^b^	7449.8^a^
P	7486.2^b^	7438.4^ab^	4930.9^b^	8570.8^a^
I	4837.3^c^	4946.3^b^	3251.6^c^	3209.1^b^
LSD	948.0	3314.4	1197.6	886.6
P value	<0.0001	0.4018	0.0003	<0.0001
CV	21.2	21.3	29.3	29.4

I: Inoculant; P: Phosphorus; M: Manure; S: Supplementary irrigation. Within each.

Location and for each variable, means followed by the same letter are not.

Significantly different according to LSD (P ≤ 0.05).

At Lere in 2017, the application of input bundle S + P + M + I increased total dry matter by 39, 58, and 50% over the input bundles P + M + I, P + I, and P, respectively. The differences were not significant among input bundles P + M + I, P + I, and P for total dry matter. There were no significant differences in 2018 among the input bundles S + P + M + I, P + M + I, P + I, and P. In comparison with other input bundles in both years, the I treatment produced the lowest total dry matter (Table 3). The treatment S + P + M + I consistently gave the highest grain yield over the other input bundles in both locations (Table 4). The treatment with only *Bradyrhizobium* produced the lowest grain yield in both locations. In Kano, the application in 2017 of input bundle S + P + M + I increased grain yield by 41, 62, and 72% over that of P + M + I, P + I, and P, respectively. The application of P + M + I produced grain yields that were 15 and 22% higher than bundles P + I and P. There was no significant difference between the grain yields produced by bundles P + I and P.

**Table 4:** Effect of input bundles on grain yield of soybean at Kano and Lere in 2017 and 2018 rainy seasons.

Treatments	Kano		Lere	
	Grain Yield (kg ha^−1^)		Grain Yield (kg ha^−1^)	
	2017	2018	2017	2018
S + P + M + I	3363.2^a^	3297.6^a^	2507.5^a^	2653.3^a^
P + M + I	2383.5^b^	2683.7^ab^	1713.2^b^	2347.3^b^
P + I	2080.4^c^	2366.0^b^	1442.9^c^	2074.2^b^
P	1953.0^c^	2097.6^b^	1550.6^c^	2099.6^b^
I	1048.3^d^	1013.8^c^	843.9^d^	974.9^c^
LSD	265.46	674.78	116.33	299.41
P value	<0.0001	0.0003	<0.0001	<0.0001
CV	36.1	36.5	34.7	29.7

I: Inoculant; P: Phosphorus; M: Manure; S: Supplementary irrigation. Within.

Each location and for each variable, means followed by the same letter are not.

Significantly different according to LSD (P ≤ 0.05).

In Lere, the input bundle S + P + M + I in 2017 produced a grain yield that was 46% more than the input bundle P + M + I, 74% more than P + I, and 62% more than P. The application of input bundle P + M + I produced grain yields that were 19 and 11% higher than P + I and P. In 2018, the application of input bundle S + P + M + I produced grain yields that were 13, 28, and 26%, respectively, more than input bundles P + M + I, P + I, and P. The differences among input bundles P + M + I, P + I, and P for grain yield were not significant. The application of input bundle P produced a mean grain yield that was 101% higher than I (Table 4).

### 3.4 Economics of input bundles

Table 5 shows the analysis of production economics on input bundles. In Kano, the application of input bundle S + P + M + I resulted in the highest MGM of $523.3 ha^-1^ (Table 5). The MGM decreased by 3, 19, 38, and 413%, respectively, for input bundles P + M + I, P + I, P and I. Similarly, S + P + M + I gave the highest MGM of $406.2 ha^-1^ in Lere. The MGM decreased by 8% for P + M + I, 28% for P + I, 16% for P, and 305% for I. The combined application in Kano of phosphorus, manure, and Rhizobia inoculant without supplementary irrigation (P + M + I) gave the highest BCR of 1.42 followed by the input bundles P + I (1. 36), P (1.21), and S + P + M + I (0.85). In Lere, the application of P gave the highest BCR of 1.28 followed by 1.19 for P + M + I, 1.12 for P + I, and 0.85 for S + P + M + I. The treatment with only *Bradyrhizobium* gave the lowest BCR in both locations (Table 5).

**Table 5:** Economic production analysis of input bundles on soybean averaged over 2-year (2017–2018) production period at Kano and Lere.

Treatment	MGY (kg ha^−1^)		Price (US $ kg^−1^)		MGR (US $ ha^−1^)		MVC (US $ ha^−1^)		MGM (US $ ha^−1^)		BCR	
	Kano	Lere	Kano	Lere	Kano	Lere	Kano	Lere	Kano	Lere	Kano	Lere
S + P + M + I	3330.4	2580.4	0.343	0.343	1141.85	884.71	618.57	478.57	523.28	406.14	0.85	0.85
P + M + I	2533.6	2030.3	0.343	0.343	868.66	696.1	358.57	318.57	510.09	377.53	1.42	1.19
P + I	2223.2	1748.6	0.343	0.343	762.24	599.52	322.86	282.86	439.38	316.66	1.36	1.12
P	2025.3	1825.1	0.343	0.343	694.39	625.75	314.29	274.29	380.1	351.46	1.21	1.28
I	1031.1	909.4	0.343	0.343	353.52	311.79	251.43	211.43	102.09	100.37	0.41	0.47

MGY = Mean grain yield; MGR = mean gross return; MVC = mean variable cost; MGM = mean gross margin; BCR = benefit-cost ratio.

## 4 Discussion

There were consistent effects of location on the parameters measured, with soybean performance better in Kano than in Lere, despite the fact that rainfall is higher and better distributed in Lere than in Kano. This might be due to differences in the texture because soil fertility indices were better in Lere than in Kano. The high clay content in Lere may fix P in the soil and make it less available to the soybean crop because P sorption increases with increase in clay content [51]. For instance where no P was applied, dry matter and grain yield was signficantly higher in Kano than in Lere suggesting that residual P was readily available to the soybean crop in Kano. The variety used in this study was an extra-early-maturing soybean variety that was able to complete its growth cycles within the season in Kano. There were inconsistent effects of the year on the parameters measured in both locations. For example, the number of nodules was higher in 2018 than in 2017 in Kano, but in Lere, the number of nodules was higher in 2017 than in 2018. This might be due to changes in the site within each location during the establishment of the trials and differences in rainfall between years.

The use of input bundles that contain supplementary irrigation, manure, P, and Rhizobia inoculant, or those that contain P, manure, and inoculant, or P and inoculant increased the performance of the crop in terms of the number of nodules per plant in both locations compared with inoculant only. The increase in nodulation with the S + P + M + I bundle could be due to a reduction in water limitation as a result of supplementary irrigation, which enhanced the symbiotic relationship. Our result supports the findings of Ampofo et al. [52], who reported a significant increase in the number of nodules by 23% when soybean was irrigated in the Guinea savanna zone of Ghana. Abdelhamid et al. [53] also reported a decrease in number of nodules under moisture-stressed conditions in Egypt.

Although the application of P improved legume response to inoculation in P-deficient soils [54], the results showed that application of P + I and P produced lower numbers of nodules than the combined application of P + M + I. This could be attributed to the application of organic manure that enhanced the survival of soil Rhizobia by the addition of organic carbon which improves crop response to inoculation [55]. This result supports the findings of Ulzen et al. [56], who reported an increase in nodule numbers in cowpea with the addition of *Bradyrhizobium* inoculant, P, and cattle manure in Ghana.

The application of inoculant alone did not influence nodulation and grain yield. This could be attributed to the very low soil P content of the study sites which limited soybean response to rhizobium inoculation without P application, which suggests that inoculation with *Bradyrhizobium* should be complemented with the application of P. This assertion is supported by the earlier findings of Kamara et al. [18,19], who report that leguminous crops require an adequate supply of P for growth and N fixation and their effectiveness in soil improvement can be hindered by P deficiency in soil. Soybean nodulation was enhanced when inoculant was applied in combination with P. This could be attributed to the adequate supply of P that regulates the BNF process and enhanced nodule and energy-generating metabolism and nodule formation thereby increasing N fixation and nodule biomass of soybean [57,58]. This corroborate the findings of [[58], [59], [60], [61]] who reported increase in nodulation parameters such as, nodule number, nodule weight and nodule biomass in response to P fertilization.

The combined use of supplementary irrigation, application of P, and manure along with inoculation with *Bradyrhizobium,* consistently increased soybean dry matter and yield compared with other treatments across years and locations. The higher dry matter and grain yield obtained from input bundle S + P + M + I as compared with P + M + I suggested that rainfall was not sufficient in the study areas. The application of supplementary irrigation might have increased available soil moisture during the vegetative and reproductive growth of the plants. The increase in yield could be attributed to increase in water uptake at the reproductive stage indicating that supplementary irrigation stimulated greater water uptake from the profile, resulting in more vigorous growth and greater podding [62]. This increase in yield and dry matter of soybean with the input bundle S + P + M + I is consistent with other findings [34,62]. Bhatia et al. [63] reported a very large gap between water non-limiting and water-limiting potential yields at locations in India where crop season rainfall was low.

The supplementary irrigation in the two years of the study might have ensured good decomposition and mineralization of the poultry manure, thus releasing the nutrients required by soybean. These findings are similar to those of Ansart et al. [64] who reported that irrigation applied at different growth stages gave maximal seed yield which was associated with better growth and yield components.Water stress reduces the grain-filling period and the transfer of assimilates into the grain of soybean plants [65]. This result also supported the findings of [34,62,66,67], who observe positive yield responses from soybean when irrigated.

Although greater yield response was observed with the treatment containing supplementary irrigation, the combined application of P + M + I without supplementary irrigation also significantly increased grain yield compared with other treatments. The increase in yield could be from the complementary role played by each factor. For example, the organic manure could have provided the needed carbon and other micronutrients to enable the applied *Bradyrhizobium* strain to survive and support the general growth of the plant, while P could have provided the required energy for N fixation and supported the overall growth of the host plant [56,68]. Additionally, the study area had a low amount of the nutrients required for plant growth (especially N and P); therefore, the improved response of soybean to the amendments could be as a result of the higher total dry matter produced from the combined effects of Rhizobia, P-fertilizer, and organic manure as reported by Ulzen et al. [23]. Previous studies have reported increases in soybean yields with improved varieties [69], P fertilizer application [17,70], inoculation with *Bradyrhizobium* [56,71,72], or combination of them [23]. Ulzen et al. [73] reported that the combined application of *Bradyrhizobium* inoculant, P, and organic manure could increase the grain yield of cowpea by up to 327%. Our result agrees with the findings of Ulzen et al. [23], who also report that the combined application of Rhizobia inoculant, P-fertilizer, and organic manure significantly increased soybean grain yields compared with the I treatment. The similar grain yield response observed from treatments containing P + I and that of P alone, as well as the lowest grain yield obtained with application of I alone, showed the importance of P for soybean growth and yield. In addition, the soils may contain high levels of indigenous Rhizobia from the widespread cultivation of soybean in the study area. Several authors have reported significant yield increases with the application of P to soybean [7,19,[74], [75], [76]]. This result is in contrast to results obtained by Adjei-Nsiah et al. [73], who showed that the application of Rhizobia inoculant alone produced a grain yield increase of 275 kg ha^−1^ in northern Ghana. Our results are also contrary to the findings of Ronner et al. [12,77], who reported an increase in grain yield of soybean from the application of inoculant alone.

The combined application of P and manure along with inoculation with *Bradyrhizobium*, without supplementary irrigation was the most profitable at Kano with BCR of 1.42, representing an increase of 246.3% over the I only treatment. At Lere, the P only treatment was the most profitable with BCR of 1.28 representing a 172.3% increase over the I only treatment that was not profitable as the BCR was <1. These differences in BCR could be attributed to the components of production as the profitability potential of any crop production depends on yield and output price [5]. These results are similar to the findings of [40] who reported a minimum BCR ≥1 as profitable for adoption of input in soybean. Our result is also in agreement with the findings of Awuni et al. [5] who reported a BCR of 1.8 for P only. Ulzen et al. [23] also reported higher BCR with the combined application of Rhizobia inoculant, P-fertilizer, and fertisoil organic manure in a soybean inoculation study in northern Ghana. Our results are contrary to those of Masso et al. [78] and Ronner et al. [12], who reported that about 60–95% of farmers who used inoculant alone in soybean production in Nigeria achieve economic benefit. Masso et al. [78] also reported that inoculant application alone was financially rewarding for farmers in northern Ghana. The low BCR of input bundle S + P + M + I is attributed to high MVC as a result of the high cost of supplementary irrigation. Differences in BCR results could also be attributed to several factors, including spatial differences in soil types or fertility that limited crop yield [39]. The combined application of phosphorus, manure, and inoculant is an effective means of increasing soybean grain yields on smallholder farms. However, the difference in grain yields might be due to varying factors in the soil, environment, and management.

## 5 Limitations of the study

One of the limitations of this study is the lack of an absolute or biological control treatment. In soil input trials, a control treatment is used to measure the contribution of inherent soil fertility to the response parameter. The increases in grain yield due to any of the treatment combinations may also be due to the additional influence of native soil fertility. In future studies, a control treatment which copies the farmer's usual level of the experimental variables, should be used for comparison. While the carbon and nitrogen contents of the manure used were quantified, C:N ratio, lignin and polyphenols were not measured. In addition, the rhizobium load of the soil was not determined. While characterization of the manure and determination of rhizobium were considered outisde the scope of our study, their non estimation is major limitation to our study and should be considered during the design of future trials involving the use of manure and rhizobium inoculants.

## 6 Conclusions

Although the combined application of nutrient inputs under supplementary irrigation produced the highest grain yield, it was not profitable due to high cost of supplementary irrigation. Also, the combined application of nutrient inputs without supplementary irrigation and application of P alone significantly increased soybean grain yield. The rhizobium inoculation alone had the least grain yield and was the least profitable input bundle. Additionally, the input bundles P + M + I and P gave higher BCR with input bundle P + M + I giving the highest in Kano and P only giving the highest in Lere, suggesting that the cultivation of soybean in Nigeria as a commercial crop could significantly increase the yield and income of farmers in the savannas if fertilizer P is applied in combination with *Bradyrhizobium* inoculation and (where possible) the addition of manure. Our results showed that supplementary irrigation of soybean along with other inputs was not profitable in the study area. Our results show that the production of soybean requires a shift from low input use to an integrated input use, which requires the use of P fertilizer in combination with *Bradyrhizobium* inoculation and manure to increase soybean yield.

## Funding

The research was funded by the 10.13039/100000865Bill & Melinda Gates Foundation through the project Putting Nitrogen Fixation to Use for Smallholder farmers in Africa with Grant No. OPP1020032.

## Data availability statement

The data that support the findings of this study are available from the corresponding author upon reasonable request.

## CRediT authorship contribution statement

**J.F. Bebeley:** Writing – review & editing, Writing – original draft, Visualization, Validation, Conceptualization, Data curation, Formal analysis, Investigation, Methodology. **A.Y. Kamara:** Writing – review & editing, Writing – original draft, Visualization, Validation, Supervision, Software, Resources, Project administration, Methodology, Investigation, Funding acquisition, Formal analysis, Data curation, Conceptualization. **J.M. Jibrin:** Writing – review & editing, Writing – original draft, Supervision, Resources, Conceptualization, Funding acquisition, Project administration. **A.I. Tofa:** Writing – original draft, Visualization, Validation, Writing – review & editing. **R. Solomon:** Validation, Methodology, Investigation. **N. Kamai:** Writing – review & editing, Supervision, Funding acquisition.

## Declaration of competing interest

The authors declare the following financial interests/personal relationships which may be considered as potential competing interests:

Dr. Alpha Yaya Kamara reports financial support was provided by 10.13039/100000865Bill & Melinda Gates Foundation. If there are other authors, they declare that they have no known competing financial interests or personal relationships that could have appeared to influence the work reported in this paper.
